# Reproducibility and repeatability of measuring the electrical impedance of the pregnant human cervix-the effect of probe size and applied pressure

**DOI:** 10.1186/1475-925X-8-10

**Published:** 2009-06-17

**Authors:** Roobin P Jokhi, Vidita V Ghule, Brian H Brown, Dilly OC Anumba

**Affiliations:** 1 Academic Unit of Reproductive and Developmental Medicine, University of Sheffield and Sheffield Teaching Hospitals NHS Trust, Sheffield, UK; 2 Department of Medical Physics and Engineering, University of Sheffield and Sheffield Teaching Hospitals NHS Trust, Sheffield, UK

## Abstract

**Background:**

The utility of cervical electrical impedance spectroscopy (EIS) as a diagnostic tool is being investigated in clinical trials. We sought to assess the reliability of two different sizes of tetrapolar probes used in measuring cervical impedance.

**Methods:**

Cervical transfer impedance was measured at 14 frequencies between 76 and 625 000 Hz from 11 pregnant subjects at term. Repeated measurements were taken with two probes (3 mm and 12 mm diameter) applied softly (approximately 0.7 Newton of force), and firmly (approximately 2.2 Newton) to the surface of the cervix by two observers. The intra-class correlation coefficient (ICC), coefficient of variation (CV) and repeatability standard deviations (SD) were derived from these measurements and compared.

**Results:**

Measurements taken by one observer were highly repeatable for both probes as demonstrated by high ICC and low CV values. Probe performance was improved further by firm application. Firm application of the 3 mm probe resulted in ICC values that ranged from 0.936 to 0.986 (p = 0.0001) and CV values between 1.0 and 3.4%. Firm pressure with the 12 mm probe resulted in ICC values that ranged between 0.914 and 0.988 (p = 0.0001) with CV values between 0.7 and 2.1%. In addition, the repeatability SD was low across all frequencies implying that there was low intra-observer variability. Measurements taken by 2 observers with firm application of the 12 mm probe demonstrated moderate reproducibility between 9.8 and 156 kHz, the frequency range in which previous clinical studies have shown predictive association between high cervical resistivity and vaginal delivery: ICC values ranged between 0.528 and 0.638 (p < 0.05), CV values were between 3.3 and 5.2% and reproducibility SD values were also low. In contrast the 3 mm probe demonstrated poor reproducibility at all study frequencies.

**Conclusion:**

Measuring cervical resistivity by a single observer with both the 3 and 12 mm probes is highly repeatable whilst inter-observer reproducibility is poor with the 3 mm probe but moderately good when the 12 mm probe is firmly applied to the cervix in the frequency range 9.8 to 156 kHz, consistent with our observations of probe performance in clinical trials.

## Background

The capacity of biological tissues to conduct electrical current is a function of the electrical frequency applied and the resistive and capacitive properties of cellular and non-cellular tissue components [1,2]. At frequencies of a few kHz to 1 MHz, cell structures are the main determinant of tissue impedance [1]. These principles form the basis of the technique of electrical impedance spectroscopy (EIS). EIS has been employed to study several human organ systems including the cervix both *in vitro *[3,4] and *in vivo*, and in non-pregnant [1,5] and pregnant women [6-8].

Significant differences in tissue electrical resistivity have been described between women with normal cervical epithelium and those with intra-epithelial neoplasia using a measuring probe with a diameter of 3 mm [1,5]. As well as describing cervical resistivity during each pregnancy trimester [6], we have recently determined that cervical resistivity measured at 9.8 – 78 kHz with a 12 mm probe has limited predictive value for vaginal delivery in women undergoing induction of labour [9]. If the changes associated with pre-labour cervical remodelling were shown to be accurately captured by measuring cervical tissue electrical resistivity this technique could find clinical application for the prompt prediction and diagnosis of preterm labour. Such advance may enable earlier therapeutic intervention to prevent preterm birth, the principal cause of perinatal death and childhood handicap worldwide.

Computer Finite Element modelling has shown that larger diameter probes with a wide inter-electrode distance, such as the 12 mm probe, enable a higher fraction of injected current to pass into the cervical stroma [6] thereby detecting changes in these deeper tissue layers. Modelling studies have also shown that surface epitheliium determines electrical resistivity at low frequencies whilst both epithelial and sub-epithelial stromal tissue characteristics inform resistivity at higher frequencies [4]. These considerations inform current trials of the 3 and 12 mm probes for pre-cancer cervical screening and pre-labour prediction studies respectively.

Data derived from a measuring device should show close agreement between readings taken by the same operator (repeatability) and between operators (reproducibility) [10] if such device is to find clinical utility. Several factors are known to influence the reliability of cervical resistivity measurement. Increasing the force of application of the measurement probe on tissue has been shown *in vitro *to increase tissue resistivity values [11]. The thickness of the mucus covering on the cervix also appears to affect cervical resistivity [12]. Measuring electrical impedance across epithelial tissue boundaries, such as the squamo-columnar junction of the cervix, may compromise its ability to discriminate between tissue types [12]. We have also shown that the distance between the injecting and sensing electrodes of the measurement probe greatly alters the magnitude of tissue resistivity values obtained [6] and influences predictive clinical ability [9]. However no study has described the reliability of cervical EIS measured by the various probe configurations currently employed in research and clinical studies.

The purpose of this study was to determine the reproducibility and repeatability of cervical resistivity measurements derived from two separate tetrapolar probes, measuring 3 mm and 12 mm in diameter, applied with two different degrees of force on the cervix – soft and firm – by two independent observers. These studies are with a view to enhancing future probe design and performance.

## Methods

### Subjects

Eleven women were studied at the time of elective caesarean section at 38–40 weeks gestation. Written informed consent was obtained from each participant. The study was approved by the South Sheffield Research Ethics Committee. Participants were excluded if they had any of the following: previous cervical surgery, multiple pregnancy, ruptured fetal membranes prior to Caesarean section, reproductive birth defects, or cervical dilatation > 3 cm.

### Cervical EIS studies

**Figure 1:**
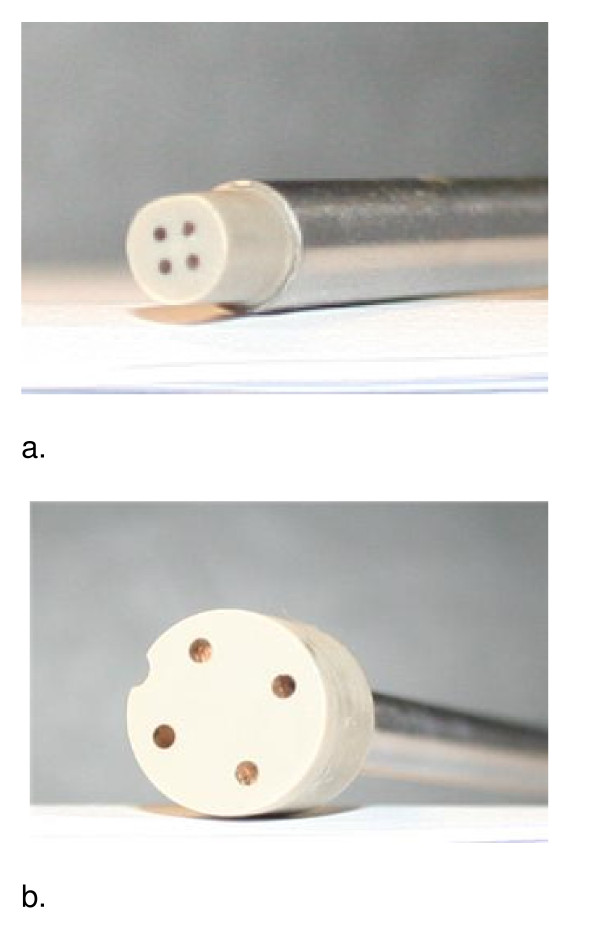
**Electrode configuration of tetrapolar probes used in the study**. 1a. Sheffield 3 mm probe: pitch circle diameter 2 mm, electrode separation (between centres) 1.41 mm, electrode diameter 0.6 mm, peak current 3.0 μA. 1b. Sheffield 12 mm probe: pitch circle diameter 8.5 mm, electrode separation (between centres) 6.01 mm, electrode diameter 1.50 mm, peak current 12.5 μA.

**Figure 2:**
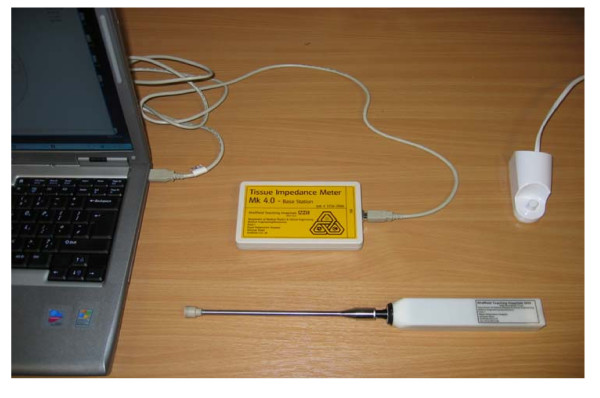
**Setup of Sheffield Mark IV Impedance Measurement system**.

The impedance measuring device consists of tetrapolar probes of different sizes (3 mm and 12 mm, Figure 1) that attach to a single channel electrical impedance measurement system (the Sheffield Tissue Impedance Meter Mk 4.0, University of Sheffield, Figure 2).

Cervical resistivity was measured with the 3 and 12 mm probes by two observers conversant with the technique. Prior to these experiments an electronic balance was used to pre-calibrate the two forces of application to be employed in a modification of the method described by Gonzalez-Correa [11]. These forces equated to approximately 0.7 Newton ("soft"), and 2.2 Newton ("firm"). The latter approximates to the degree of force routinely employed by us in our clinical experiments. Prior to each experiment, the two observers undertook a balance calibration to ensure that the force applied to the measuring probe was consistent between observers and equated to the approximate force of application being studied. In preparation for Caesarean section, each participant was anaesthetised by means of spinal block, positioned in lithotomy, and a Cusco's speculum used to gently expose the cervix. Using a cotton swab, any thick vaginal mucus obscuring the view obtained was gently removed avoiding contact with the epithelial surface.

For each probe, measurements were taken by each of the two operators from the 12 o'clock position on the anterior lip of the cervix. Each observer took 2 measurements 1–2 minutes apart applying the probe to the cervix with each of the two pre-determined degrees of force. The order of the probes was randomised to avoid systematic bias. The impedance measuring device is connected wirelessly to a computer with a Matlab^® ^software interface (The Mathworks Inc., Natick, MA, USA) for data capture and display. The basic design of this measurement system *in vivo *has been described previously [1]. Transfer impedance values measured in ohms (Ω) were simultaneously obtained at 14 electrical frequencies ranging from 76 Hz to 625 kHz increasing in octave steps and stored in ASCII format.

### Statistical analysis

The data were initially tested for normality of distribution by means of the Kologorov-Smirnov test. As this suggested that the data were not normally distributed, a logarithmic transformation was applied to all the data before statistical analysis. For each, repeatability (intra-observer variability) and reproducibility (inter-observer variability) measures were derived for each of the 14 frequencies studied at the two forces of application: the intra-class correlation coefficient (ICC), the coefficient of variation (CV), the repeatability and reproducibility standard deviations and 95% limits of agreement of repeat measurements, all of which methods have been extensively employed in the comparison of repeated measurements between observers [13-15]. The ICC is a method of measuring inter-rater reliability and is computed from a one-way analysis of variance (ANOVA) on the log transformed data as described by Shrout and Fleiss [16]. The effect of the following variables on the repeatability and reproducibility of EIS measurements were investigated: observer, probe size and application force. Intra-class correlation coefficients were determined using one-way random single measure for intra-rater analysis and two-way mixed model, absolute agreement definition for inter-rater analysis. Reliability was regarded as excellent if ICC > 0.75, fair to good if 0.4 ≤ ICC ≥ 0.75, and poor if ICC ≤ 0.4 [17]. The coefficient of variation measures the dispersion of a distribution and is useful in comparing data sets with very different means as occurs in EIS measurements between individuals. The CV was derived as the ratio of the standard deviation of each group of measurements to the mean [18]. The repeatability and reproducibility standard deviations were derived from Bland-Altman plots [19] which also summarised the limits of agreement between: a) 2 measurements taken by each observer at a defined transducer pressure and b) 2 measurements taken by two observers at a defined transducer pressure [19]. When comparing data derived for soft pressure to that for firm pressure for each probe, a two-tailed paired Student t test was also performed.

Statistical analysis was performed using SPSS for Windows (version 15.0, SPSS Inc, Chicago IL) and the MedCalc (version 9 Mariakerke, Belgium) statistical packages.

## Results

The mean (SE) age of study participants was 31(3.3) yrs and median (range) gestation was 271 (267 – 276) days.

### Repeatability of cervical resistivity measurements

**Figure 3:**
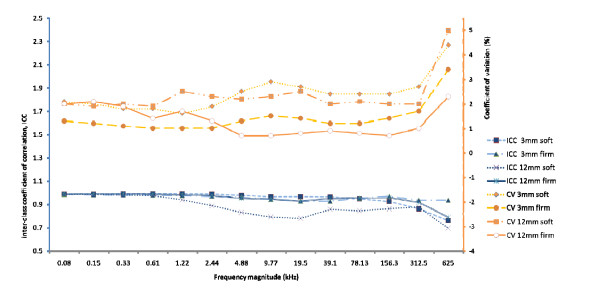
**Intra-class correlation coefficients (ICC) and coefficient of variation (CV) obtained by one observer**. Intra-class correlation coefficients (ICC) and coefficient of variation (CV) of cervical resistivity values obtained by a repeat measurement by one observer applying soft (0.8 Newton) and firm (2.2 Newton) force on the cervix for each of the 2 probes studied.

Figure 3 illustrates the ICC and CV for resistivity measurements obtained by Observer 1 for each of the two study probes. Applying soft pressure the 3 mm probe demonstrated excellent ICC that ranged from 0.862 to 0.991 (p = 0.0001) with low CV ranging between 1.6 and 2.9% for the first 13 frequencies. The ICC for frequency 14 (625 kHz) was 0.764 (p = 0.002) with a CV of 4.4%. When the probe was firmly applied to the cervix the ICC values were excellent and ranged from 0.936 to 0.986 (p = 0.0001) with CV values between 1.0 and 3.4%.

Applying soft pressure the 12 mm probe demonstrated ICC values that ranged between 0.782 and 0.986 (p = 0.001) and CV of 1.9 to 2.5% for the first 13 frequencies and ICC of 0.695 (p ≤ 0.006) with a CV of 5% for frequency 14 (625 kHz). Firmer application of the probe improved the repeatability with ICC values of 0.914 to 0.988 (p = 0.0001) and CV values of 0.7 to 2.1% for the first 13 frequencies. The ICC for frequency 14 (625 kHz) was 0.792 (p = 0.001) with a CV of 2.3%.

**Figure 4:**
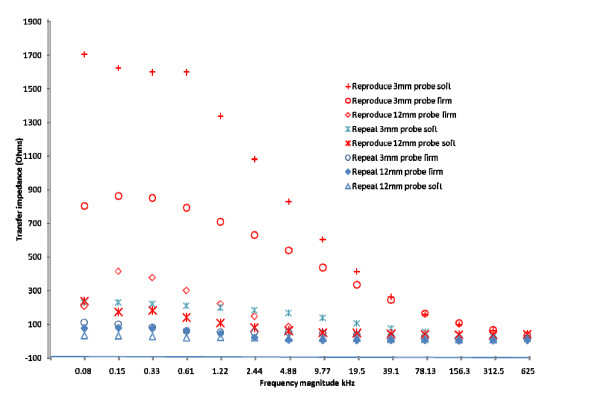
**Repeatability and reproducibility standard deviations for cervical resistivity by probe and force of application**. Repeatability (one observer) and reproducibility (two observers) standard deviations for cervical resistivity derived by both the 3 mm and 12 mm probes applied to the cervix softly (0.8 Newton) and firmly (2.2 Newton).

The repeatability standard deviations for both probes are depicted in Figure 4, whilst the 95% confidence limits of agreement of repeat measurements by each observer are summarised in Tables 1 and 2.

**Table 1:** Summary of intra- and inter-observer limits of agreement of resistivity values (Ohms) derived at 14 frequencies by firm pressure of the 3 mm probe on cervix (n = 11).

	**Repeatability**								**Reproducibility**			
	
	**Observer 1**				**Observer 2**				**Inter-observer agreement**			
**Test frequency**	**Mean 1^st ^test**	**Mean 2^nd ^test**	**Mean difference**	**Limits of agreement**	**Mean 1^st ^test**	**Mean 2^nd ^test**	**Mean difference**	**Limits of agreement**	**Observer 1**	**Observer 2**	**Mean difference**	**Limits of agreement**

**1**	872.4	877.4	-5	-222 to 212	889.9	907.3	-17.4	-180 to 145	872.4	889.9	-17	-1595 to 1560

**2**	923.9	918	6	-190 to 202	947.2	969.9	-22.6	-205 to 160	923.9	947.2	-23	-1717 to 1670

**3**	812.0	896.8	15	-141 to 171	940.7	963.2	-22.5	-209 to 164	912.0	940.7	-29	-1700 to 1642

**4**	863.0	839.0	24	-95 to 142	891.6	915.5	-23.9	-216 to 167	863.0	891.6	-26	-1419 to 1366

**5**	784.5	755.1	29	-72 to 130	810.7	835.8	-25.2	-223 to 173	784.5	810.7	-26	-1419 to 1366

**6**	718.9	684.5	34	-66 to 135	733.9	761.5	-27.6	-233 to 178	718.9	733.9	-15	-1253 to 1223

**7**	637.3	605.3	32	-69 to 133	635.6	659.9	-24.4	-239 to 190	637.3	635.6	-2	-1058 to 1062

**8**	552.1	524.9	27	-62 to 116	535.5	557.6	-22.0	-225 to 182	552.1	535.5	17	-844 to 878

**9**	468.5	451.0	17	-56 to 91	445.8	464.9	-19.1	-196 to 158	468.5	445.8	23	-636 to 681

**10**	389.9	379.4	11	-40 to 61	368.8	384.3	-15.5	-165 to 134	389.9	368.8	21	-460 to 502

**11**	316.7	316.1	1	-44 to 45	300.0	312.3	-12.3	-123 to 98	316.7	300.0	17	-307 to 340

**12**	251.7	252.6	-1	-32 to 30	233.5	243.9	-10.4	-94 to 73	251.7	233.5	18	-195 to 230

**13**	177.8	182.3	-5	-30 to 21	168.1	175.0	-6.8	-69 to 56	177.8	168.1	10	-122 to 141

**14**	91.5	102.0	-11	-39 to 17	99.1	98.8	0.3	-27 to 28	91.5	99.1	-8	-77 to 62

**Table 2:** Summary of intra- and inter-observer limits of agreement of resistivity values (Ohms) derived at 14 frequencies by firm pressure of the 12 mm probe on cervix (n = 11).

	**Repeatability**								**Reproducibility**			
	
	**Observer 1**				**Observer 2**				**Inter-observer agreement**			
**Test frequency**	**Mean 1^st ^test**	**Mean 2^nd ^test**	**Mean difference**	**Limits of agreement**	**Mean 1^st ^test**	**Mean 2^nd ^test**	**Mean difference**	**Limits of agreement**	**Observer 1**	**Observer 2**	**Mean difference**	**Limits of agreement**

**1**	526.9	539.3	-12	-162 to 127	350.0	366.3	-16	-108 to 76	526.9	539.3	204	-626 to 1033

**2**	499.6	512.9	-13	-167 to 140	347.2	362.6	-15	-92 to 61	499.6	512.9	179	-636 to 993

**3**	438.7	453.7	-15	-161 to 131	317.6	329.8	-12	70 to 45	438.7	453.7	145	-594 to 884

**4**	352.2	365.5	-14	-133 to 106	268.2	275.4	-7	-45 to 30	352.2	365.5	103	-487 to 693

**5**	266.0	278.1	-12	-100 to 75	212.6	219.0	-7	-33 to 20	266.0	278.1	68	-368 to 503

**6**	210.3	215.5	-5	-46 to 35	175.8	181.2	-5	-31 to 20	210.3	215.5	44	-244 to 235

**7**	178.8	181.9	-3	-17 to 10	151.9	157.4	-6	-28 to 17	178.8	181.9	36	-135 to 206

**8**	157.9	158.5	-1	-10 to 9	140.8	145.0	-4	-26 to 17	157.9	158.5	25	-72 to 121

**9**	145.6	146.3	-1	-11 to 9	134.1	136.8	-3	-22 to 16	145.6	146.3	19	-50 to 87

**10**	136.8	136.5	0.5	-10 to 11	128.2	130.8	-3	-22 to 17	136.8	136.5	15	-40 to 71

**11**	129.0	127.7	1	-9 to 11	123.1	124.6	-1.5	-19 to 15	129.0	127.7	12	-42 to 66

**12**	118.9	117.5	1.5	-5 to 8	112.9	113.2	-0.2	-13 to 13	118.9	117.5	11	-31 to 53

**13**	96.6	96.6	0.1	-8 to 8	95.2	95.9	-0.7	-7 to 5	96.6	96.6	5	-32 to 43

**14**	54.6	54.4	0.5	-9 to 10	55.0	54.9	0.1	-11 to 11	54.6	54.4	-1	-34 to 33

### Effect of application force on cervical resistivity values obtained by one observer

**Figure 5:**
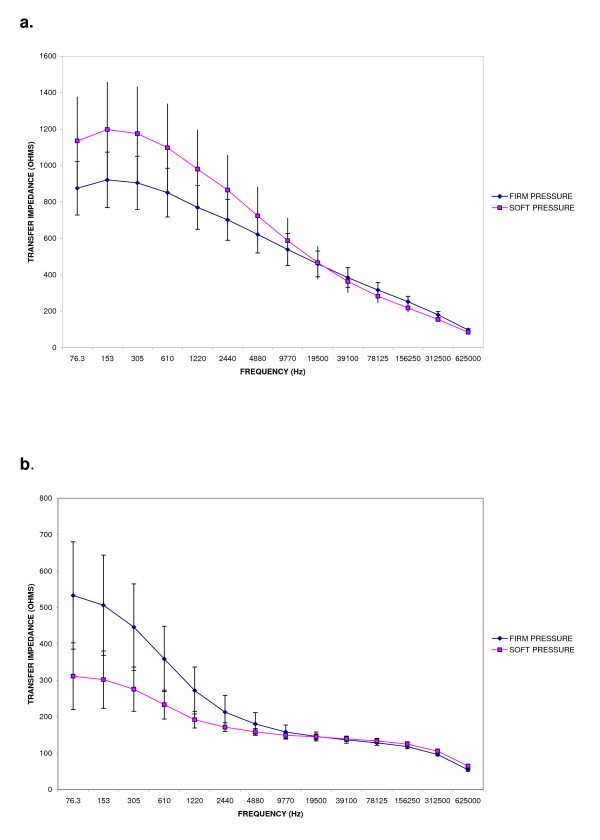
**Variability of cervical resistivity by application pressure for each probe studied by Observer 1**. **a**: Mean (SE) cervical resistivity values across 14 measured frequencies using the 3 mm probe by force of probe application to the cervix. **b**: Mean (SE) cervical resistivity values across 14 measured frequencies using the 12 mm probe by force of probe application to the cervix.

Cervical resistivity values obtained by one observer when the force of probe pressure on the cervix was changed from soft to firm are summarised for the 2 study probes in Figure 5. No differences in resistivity by variation in application force attained statistical significance at any frequency for either probe. However a trend towards a reduction in resistivity with firm application of the 3 mm probe and towards an increase in resistivity with the 12 mm probe was apparent at low frequencies ≤ 9.8 kHz (Figure 5a and 5b respectively). At frequencies above 9.8 kHz pressure had little or no affect on cervical resistivity.

### Reproducibility of cervical resistivity measurements

**Figure 6:**
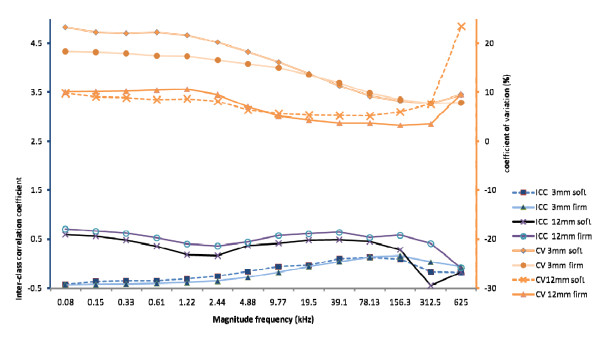
**Intra-class correlation coefficients (ICC) and coefficient of variation (CV) obtained by two observers**. Intra-class correlation coefficients and coefficient of variation of cervical resistivity values obtained by two observers taking measurements with soft (0.8 Newton) and then firm (2.2 Newton) probe application on the cervix.

Figure 6 illustrates the ICC and CV for resistivity measurements taken by two observers with soft and firm application of the two study probes on the cervix.

The 12 mm probe demonstrated better inter-observer reproducibility of measurements than the 3 mm probe as shown by higher ICC values and lower CV across frequencies. For the 12 mm probe the moderate reproducibility with soft application (ICC 0.410 to 0.553 P < 0.05, CV 5.2 to 9.1%) improved with firm force of application, achieving best values between the frequencies 9.8 and 156 kHz (ICC 0.528 to 0.638 p < 0.05, CV 3.3 to 5.2%). In contrast the 3 mm probe demonstrated poor reproducibility both with soft (ICC -0.431 to 0.102, p > 0.05, CV 8.2 to 23.3%) and with firm force (ICC -0.434 to 0.157, p > 0.05, CV 7.7 – 18.4%).

Figure 4 summarises both the repeatability and reproducibility standard deviations for both probes at the two levels of force studied. This illustrates the poor reproducibility but good repeatability of measurements obtained from the 3 mm probe across different force applications, and the superior reproducibility of the 12 mm probe which showed good intra-observer repeatability. The overall limits of agreement of cervical electrical resistivity measurement by examiner and by examination are summarised in Table 1 (3 mm probe) and Table 2 (12 mm) probe.

## Discussion

Ongoing clinical trials seek to assess the prediction of labour outcome by cervical EIS determinations but the accuracy of this technique for investigating the pregnant human cervix has not been systematically assessed. This study quantifies the reliability of the measurement of human cervical electrical resistivity using two tetrapolar probes of different sizes (3 mm and 12 mm) and varying the force of application of the probe on the cervix. We have confirmed that this measurement is highly reliable when repeated by one observer employing either of the two probes. We have also demonstrated that measurements by two observers are poorly reproducible for the 3 mm probe but highly so for the 12 mm probe, across all frequencies but more so at ≥ 9.8 kHz. We have shown that soft or firm application of the probe on the cervix did not appear to significantly affect resistivity values obtained by either probe. However the reliability of measurements appears better when the measuring probe is firmly applied to the cervix. Overall, tissue resistivity seemed most reproducible when the 12 mm probe is firmly applied to the cervix and readings are taken between the frequencies 9.8 – 156 kHz.

We have used several different statistical measures of reliability to summarise our observations as individual measures have inherent limitations and pitfalls [14] which are summarised elsewhere [15,20]. In the final analysis, however, it is recognised that the most reliable measurements (showing good repeatability and reproducibility) are those that demonstrate high ICC as well as low CV values [14,15] and narrow limits of agreement [19]. We have therefore based our overall conclusions on these considerations.

Our observations that application force did not appear to significantly affect tissue resistivity readings by either probe studied is somewhat at variance with observations *in vitro *using extirpated animal and human tissue specimens which demonstrated an increase in resistivity with increasing probe pressure [11]. However the latter studies were conducted on tissue placed on a hard surface such that the pressure-related increases in the resistivity readings was attributed to intercellular fluid being squeezed out whilst, at the same time, the extracellular space was reduced, and to changes in the thickness of the tissue produced by very high pressures. In contrast, in the *in vivo *situation of our experiments the pressure underneath the probe is highly likely to be much less since the cervical tissue being measured is soft, compliant and unsupported by a hard surface. However our limited sample size may have been under-powered to detect any small effects of force and pressure on tissue resistivity values. Consistent with this possibility we observed that firm application of the probe on the cervix generally improved repeatability and reproducibility of resistivity measurements as shown by better repeatability standard deviations amongst other statistical parameters.

The mechanism by which application force and pressure may alter tissue resistivity values is unclear but is likely to involve the spatial re-arrangement of the epithelial tissues. At higher frequencies any such re-arrangement resulting from an increase in probe pressure or force would be obviated by current being able to pass through the cytoplasm of the cells resulting in more similar impedance readings. The presence of mucus on the epithelial surface can act as a short-circuit resulting in lower impedance values at lower frequencies [21]. Increasing the force and pressure on the probe may then displace the high conductance mucus film on the cervix thus effectively decreasing tissue electrical conductivity and increasing resistivity. In the present studies, however, the contribution of the mucus film on the cervix to resistivity changes associated with varying application force may have been reduced by our practice of gently removing mucus from the surface of tissue.

Our findings suggest that the reliability of cervical impedance measurement is best at frequencies high enough to discriminate between tissue characteristics as well as minimise the effects of differential application force and pressure. We observed high ICC and low CV values for intra-observer repeatability for both the 3 and the 12 mm probes suggesting that measurements taken with these probes by a single observer are highly repeatable. In contrast reproducibility of measurements between two observers was poor for the 3 mm probe, as evidenced by relatively high CV values and low ICC values, but was relatively good when data was derived by firm application of the 12 mm probe especially in the frequency range 9.8 to 156 kHz. At these frequencies high ICC values were accompanied by low CV values. It is noteworthy that we have previously demonstrated in clinical studies of pregnant women prior to induction of labour an association between high resistivity values obtained in this frequency range with the 12 mm probe and delivery by caesarean section [9]. The present report suggests that the poor predictive performance of the 12 mm probe at lower electrical frequencies in our previous clinical experiments may have resulted from the poor reliability of measurements at lower study frequencies. In the present study the reduced reliability of tissue resistivity measurements by both probes at the top frequency of 625 kHz is likely due to a greater degree of signal noise, an observation also made in similar studies in other tissues [22].

In addition to the effects of application force and pressure and cervical mucus, several factors may affect the reliability of impedance measurement. The 3 mm probe is particularly sensitive to subtle changes in the cervical squamous epithelium and its application at different positions on the cervix by the same and different observers could lead to varied readings. Epithelial tissue boundaries such as the squamo-columnar junction of the cervix modify and confound the impedivity spectra obtained across them [12].

## Conclusion

Measuring cervical tissue resistivity is highly repeatable. The reproducibility of this measurement appears best when the large diameter 12 mm probe is employed, pressure is firmly applied to the probe to ensure adequate contact between probe and tissue, and data derived in the frequency range 9.8 to 156 kHz. Our observations in the current studies are consistent with our data from clinical trials which showed limited prediction of delivery by caesarean section when cervical resistivity is assessed using the 12 mm probe in women undergoing induction of labour at term. Modifications of the measuring probe to enhance reliability of measurements may increase the potential use of this device as a research and, ultimately, a clinical tool for assessing cervical remodelling prior to both term and pre-term labour.

## Competing interests

The authors declare that they have no competing interests.

## Authors' contributions

All authors have contributed to and approved the final manuscript. DOCA conceived and designed the study. RPJ and DOCA developed the methodology and RPJ and VVG acquired the data. RPJ and DOCA conducted the statistical analysis and wrote the first draft of the paper. All authors contributed to writing the manuscript and have approved the final version.
